# Hydroxyvitamin D Serum Levels are Negatively Associated with Platelet Number in a Cohort of Subjects Affected by Overweight and Obesity

**DOI:** 10.3390/nu12020474

**Published:** 2020-02-13

**Authors:** Roberta Zupo, Fabio Castellana, Rodolfo Sardone, Luisa Lampignano, Carmen Di Noia, Silvia Savastano, Gianluigi Giannelli, Giovanni De Pergola

**Affiliations:** 1 Research Unit on Frailty Phenotypes, National Institute of Gastroenterology “S. De Bellis”, Research Hospital, Castellana Grotte, 70013 Bari, Italy; zuporoberta@gmail.com (R.Z.); castellanafabio@hotmail.com (F.C.); rodolfo.sardone@irccsdebellis.it (R.S.); luisalampignano@gmail.com (L.L.); 2 Clinical Nutrition Unit, Medical Oncology, Department of Biomedical Science and Human Oncology, University of Bari, School of Medicine, Policlinic, 70124 Bari, Italy; carmen.dinoia@libero.it; 3 Department of Medicine and Surgery, Unit of Endocrinology Federico II University, Medical School of Naples, 80131 Naples, Italy; silvia.savastano@unina.it; 4 Scientific Direction, National Institute of Gastroenterology “S. De Bellis”, Research Hospital, Castellana Grotte, 70013 Bari, Italy; gianluigi.giannelli@irccsdebellis.it

**Keywords:** platelets, vitamin D, obesity

## Abstract

Background: Hypovitaminosis D and higher platelet numbers are emerging as cardiovascular risk factors, in particular in obese subjects. Methods: This observational study was aimed at investigating the relationship between platelet number and serum 25-hydroxyvitamin D (25(OH)D) levels in a cohort of individuals affected by overweight and obesity (body mass index (BMI) ≥ 25 Kg/m^2^). A sample of 341 subjects (248 women, 93 men), aged 18–71 years, taking no medication, was examined. Anthropometric, hormone, metabolic and common routine hematochemical parameters were examined and evaluated in association with platelet count and serum 25(OH)D levels. Results: Platelet numbers were inversely related to age (*p* < 0.04), 25(OH)D (*p* < 0.05) and uric acid (*p* < 0.04) levels, and directly associated with white blood cells (*p* < 0.01), Thyroid Stimulating Hormone (TSH) (*p* < 0.04), insulin levels (*p* < 0.002) and Homeostasis Model Assessment – Insulin Resistance (HOMA-IR) (*p* < 0.002). We applied statistical regression models to examine the relationship between platelet count (dependent variable) and parameters that had univariate associations with platelet numbers, showing that the association between platelet count and 25(OH)D was not confirmed. Moreover, vitamin D showed a negative independent association with BMI, diastolic blood pressure and serum insulin levels. Conclusions: This study indicates, for the first time, that vitamin D deficiency is associated with a parallel increase in platelet number, suggesting that higher platelet numbers may be one of the possible mechanisms leading to a greater cardiovascular risk in obese subjects. It also shows that vitamin D deficiency, a common condition in obesity, has independent associations with higher BMI, diastolic blood pressure and serum insulin levels.

## 1. Introduction

Vitamin D is known to be the main component of calcium homeostasis and bone mineralization [1], and a severe decrease of this vitamin leads to rickets in children, and osteomalacia and osteoporosis in adults. The 25-hydroxyvitamin D (25(OH)D) serum concentration is the best biological indicator of vitamin D status since it reflects both dietary intake and cutaneous synthesis [2]. Adequate 25(OH)D levels affect the activity of many organs and tissues in the body, and there is growing evidence of a critical role of vitamin D in the prevention of serious chronic diseases, apart from osteoporosis, osteomalacia and rickets. In fact, vitamin D receptors (VDRs) are located in a large variety of cell types including myocytes, cardiomyocytes, pancreatic beta-cells, endothelial cells, neurons, immune cells and osteoblasts [3].

Epidemiological data suggest that vitamin D deficiency, defined as plasma 25(OH)D levels below 20 ng/mL [3], is highly prevalent in the general population worldwide and may become a global risk factor for several multifactorial diseases [4]. While the lack of outdoor sun exposure and the greater use of sun protection during summertime are the main factors responsible for hypovitaminosis D, further important causes are a higher body fat mass and obesity [5,6]. In adults, hypovitaminosis D may contribute to an increased risk of cardiovascular diseases (CVDs) [6,7], cancer [8], impaired cognitive function [9] and immunological diseases (e.g., rheumatoid arthritis, autoimmune thyroiditis, etc.) [10]. Moreover, increased levels of inflammatory cytokines, such as tumor necrosis factor alpha (TNF-α) and interleukin (IL)-6, have been found in subjects with low 25(OH)D levels [11]. Interestingly, inflammation enhances oxidative stress, and this alteration contributes to the release of immature platelets from bone marrow to the circulatory system. Platelets are key elements in various systems, such as hemostasis, thrombosis, immunity and inflammation, and their activation is one of the basic indicators of atherothrombosis and CVDs [12]. A high platelet total number is a risk factor for early signs of atherosclerosis. However, we recently showed a decrease in platelet numbers in patients with thickening of the carotid artery and suggested that this may be a defensive mechanism against a further progression of atherosclerosis [13].

Platelet number is known to reflect the whole activity of releasing cytokines and promoting thrombosis [14] and several studies have previously described its activation as a hallmark feature in a context of inflammatory, autoimmune and chronic illnesses. That is the reason why many authors investigated inflammation and vitamin D status among different pathological settings. People affected by diabetes [15,16], coronary artery disease [17], endothelial dysfunction or Sjögren’s syndrome [18], which are all triggering conditions for inflammation and higher levels of inflammatory markers, have been described to exhibit poor vitamin D status. However, Park et al. recently showed an inverse association between 25(OH)D levels and platelet count in the general adult population, although they did not investigate possible differences between healthy and unhealthy subjects [19]. There is no information about whether this association exists in overweight subjects. Therefore, this study addressed the association between platelet number and 25(OH)D serum levels in patients affected by overweight and obesity.

## 2. Methods

The study population was recruited from July 2018 to June 2019 at the Outpatient Clinic of Nutrition of the Medical Oncology Unit, Department of Biomedical Sciences and Human Oncology, University of Bari, School of Medicine, Policlinic, Bari, Italy, and at the National Institute of Gastroenterology “S. de Bellis,” Research Hospital, Castellana Grotte, Italy. In total, 341 consecutive subjects were enrolled at the first examination if not taking any medication, including oral contraceptives or drugs for osteoporosis, and free from significant medical illnesses, but affected by overweight or obesity. The sample included 284 females and 93 males, aged 18–71 years. Inclusion criteria were overweight or obesity (body mass index (BMI) > 25 Kg/m^2^), and all subjects came to the Outpatients Clinic with the only aim of losing weight. Exclusion criteria were any history of endocrinological diseases (diabetes mellitus, hypo or hyperthyroidism, hypopituitarism, etc.), chronic inflammatory diseases, stable hypertension, angina pectoris, stroke, transient ischemic attack, heart infarction, congenital heart disease, malignancies, renal and liver failure, inherited thrombocytopenias and other major malignancies.

At baseline, subjects were closely examined for medical history, hormonal, metabolic and routine hematochemical parameters. We performed a clinical baseline evaluation that included extemporaneous ambulatory blood pressure (BP) and a physical assessment of body weight, body mass index (BMI) and waist circumference (WC) as anthropometric parameters. BMI was calculated by dividing body weight (Kg) by the square of height (m^2^). WC was measured at the narrowest part of the abdomen, or in the area between the tenth rib and the iliac crest (minimum circumference). The extemporaneous BP was determined in a sitting position after at least a 10-min rest, at least three different times, using an OMRON M6 automatic Blood Pressure monitor.

Blood samples were drawn between 08:00 h and 09:00 h after an overnight fast. Blood glucose, insulin, 25(OH)D, total cholesterol, high- and low-density lipoprotein (HDL, LDL) cholesterol, triglycerides, transaminases, gamma-glutamyl transferase (γGT), sodium, potassium, Red Blood Cells (RBC), White Blood Cells (WBC), platelet, iron, uric acid, TSH, FT3, FT4, creatinine serum levels and insulin resistance were assayed. Serum insulin concentrations were measured by radioimmunoassay (Behring, Scoppito, Italy). Serum 25(OH)D levels were quantified by chemiluminescence (Diasorin Inc., Stillwater, OK, USA) and all samples were analyzed in duplicate. Plasma glucose was determined using the glucose oxidase method (Sclavus, Siena, Italy), while the concentrations of plasma lipids (triglycerides, total cholesterol, HDL cholesterol) were quantified by automated colorimetric method (Hitachi; Boehringer Mannheim, Mannheim, Germany). LDL cholesterol was calculated using the Friedewald equation. Serum FT3, FT4 and TSH were measured using a competitive photometric method based on the solid phase antigen-linked technique (LIASON FT3, LIASON FT4, LIASON TSH, Dia-Sorin, Saluggia, Italy). Serum uric acid was measured by the URICASE/POD method implemented in an autoanalyzer (Boehringer Mannheim, Mannheim, Germany). Creatinine, potassium, sodium, transaminases, gamma-glutamyl transferase (γGT) and iron were measured by an automated system (UniCel Integrated Workstations DxC 660i, Beckman Coulter, Fullerton, CA, USA). Blood cell count was determined using an XT-2000i hematology analyzer (Sysmex, Dasit, Cornaredo, Italy). Insulin resistance was assessed using the Homeostasis Model Assessment – Insulin Resistance (HOMA-IR) [20].

The study protocol (ClinicalTrials.gov Identifier: NCT04133441) was approved by the Ethics Committee of the National Institute of Gastroenterology “S. De Bellis” Research Hospital, Castellana Grotte, Italy, and all participants gave prior informed consent to enrolment in accordance with the Helsinki Declaration of 1964 and subsequent revisions.

Statistical analyses of baseline variables are expressed as mean ± standard deviation (SD) for continuous variables and as proportion (%) for the frequency of categorical variables. The normality of distribution was evaluated using the Shapiro test for each sample group, and specific parametric and non-parametric tests were performed to assess the presence of significant differences. All continuous variables were assessed with Spearman’s rank correlation test, in accordance with the methodological occurrence. *p*-values less than or equal to 0.05 were considered statistically significant, with 95% confidence intervals. The association between platelets number and Vitamin D was measured with linear regression models. All models were adjusted for major confounders (age, sex, BMI, uric acid, WBC, HOMA index and TSH). Statistical analyses were performed using RStudio software, Version 1.2.5001 (RStudio, Inc., Boston, MA, USA).

## 3. Results

The entire sample consisted of 341 overweight and obese subjects. Table 1 summarizes the general, anthropometric, hormonal, metabolic and routine hematochemical parameters of the enrolled population, expressed as mean ± SD. Table 2 shows the correlation between platelet number and 25(OH)D, and the other investigated variables. Platelet number was negatively related to age (*p* < 0.04), 25(OH)D (*p* < 0.05) and uric acid (*p* < 0.04) levels. A positive association was found with blood WBC (*p* < 0.01), TSH (*p* < 0.04) and insulin (*p* < 0.002) levels and HOMA index (*p* < 0.002).

Two sets of multiple linear regressions were performed, considering platelet number as the dependent variable and 25(OH)D and other parameters that showed a significant correlation with platelet number as independent variables. The selection of confounders was made considering both the univariate significance with platelet number and major covariates such as age, BMI and sex. The first linear regression model (Table 3), using platelet number as the dependent variable, and age, HOMA, TSH, uric acid, 25(OH)D and WBC as regressors, showed that the relationship between platelet number and 25(OH)D levels was not significant. A second, adjusted linear regression model (Table 4) was performed also considering gender and BMI as regressors, confirming the lack of an independent association between platelet number and 25(OH)D levels. However, both models showed a strong positive association between WBC and platelet number. Interestingly, the second regression showed lower levels of platelets in males.

## 4. Discussion

The present study was aimed at investigating the possible link between platelet number and 25(OH)D serum levels in subjects affected by overweight or obesity.

Our data show, for the first time, that subjects with lower 25(OH)D levels have higher platelet numbers, indicating that a vitamin D deficiency in obese subjects may be predictive of a higher risk of inflammation, thrombosis and cardiovascular events in this condition. These results are in line with previous findings by Park and colleagues, that showed an inverse association between 25(OH)D levels and platelet indices in the general adult population but did not investigate possible differences between healthy and unhealthy subjects [19]. To better explain the interrelationship between obesity, vitamin D, platelet count and cardiovascular risk, it is of note that vitamin D has a direct role in regulating hematopoietic cells’ differentiation [21]. Moreover, a previous study [22] showed that vitamin D receptors modulate megakaryocytopoiesis and platelet activation, which are calcium-dependent events. Lastly, a physiological anti-thrombogenic and anti-inflammatory activity of vitamin D has also been reported [23,24]. Therefore, we believe that the consequence of events is that obesity is responsible for a decrease of vitamin D circulating concentrations [6], lower vitamin D levels are responsible for higher platelet number [21,22] and, finally, obesity is indirectly responsible for higher platelet count, thus higher cardiovascular risk.

Beyond that, the excess body weight characterizing our sample, per se, triggers a low-grade systemic inflammation by promoting the release of platelets and, possibly, proinflammatory cytokines, chemokines, adhesion molecules, tissue factor expression and endothelial activation. Thus, a combination of vitamin D deficiency, higher BMI and systemic inflammation physiologically leads to platelet activation and proliferation. Afterwards, this cascade of events also unfavorably affects endothelial function and cardiovascular health [25].

Interestingly, the inverse relationship between vitamin D and platelet number was not maintained when the effect of parameters having a significant association with platelet count (age, uric acid, WBC, TSH and HOMA index) was evaluated in a regression model. This result seems to exclude the possibility that vitamin D has a direct inhibitory effect on platelet production, even if the strong influence of gender on platelet number leaves room for a logical explanation of this loss of association. Our statistical analysis showed lower levels of platelets in men than in women and this result is in line with previous findings. The gender influence on platelet biology was shown several years ago [26] and recent studies reported a higher number of fibrinogen surface receptors and a greater ability to bind fibrinogen in women [27]. In addition, estrogens have been shown to enhance platelet production [28]. All these data are important since a direct association has been shown between platelet count and major cardiovascular risk factors [29].

Our data confirm that a lower vitamin D level is a very common condition among obese subjects [4,5], and vitamin D was independently related to higher BMI, diastolic blood pressure and insulin serum levels in this study. The close link among these unhealthy conditions is widely recognized as an integral part of obesity, particularly central obesity [29,30]. Also, our sample shows an age-related decrease in platelet number. In this regard, although many authors have suggested a possible influence of ethnicity on this relationship, the largest cross-sectional Moli-Sani Project performed in Italy found the same inverse relationship. This is likely justified by the reduction in hematopoietic stem-cell reserve during aging and by a survival advantage among subjects showing lower platelet numbers [31].

Platelet number also showed an inverse association with uric acid, and this result is in line with previous studies showing a potential role of uric acid in affecting platelet activation and reactivity [32]. Therefore, while hyperuricemia is commonly associated to a higher cardiovascular risk [33], low levels of uric acid may also be a risk factor through increased platelet numbers.

The main limitation of this study regards the selection of the sample, since all subjects were affected by overweight and obesity, thus making a control group impossible. No assessment of other platelet indices, or of sunlight exposure and evaluation of mobility status, may be weak points of this study. By contrast, a strong point is that we examined only individuals who were not taking any medication that could interfere with biomarkers assays and so there was no interference of drugs on the statistical associations.

## 5. Conclusions

The present study confirms a decrease of vitamin D in obesity and shows, for the first time, that vitamin D deficiency is related to a parallel increase of the platelet count, suggesting that the simultaneous increase of platelet number and decrease of vitamin D may be partly responsible for a higher cardiovascular risk in obese subjects.

## Figures and Tables

**Table 1:** Description of the whole sample (N = 341).

Variables	Mean ± SD (%) *	Range
Age (years)	41.26 ± 12.85	18 to 71
Gender (male)	93 (27.3)	--
BMI** (kg/m^2^)	33.2 ± 5.49	24 to 49.3
WC** (cm)	107.42 ± 12.91	79 to 150
SBP** (mmHg)	128.71 ± 15.7	90 to 180
DBP** (mmHg)	83.57 ± 11.29	56 to 120
FBG** (mg/dL)	89.46 ± 11.51	66 to 143
Insulin (mg/dL)	14.45 ± 9.08	2.4 to 67
HOMA-IR **	3.24 ± 2.16	0.52 to 14.5
Triglycerides (mg/dL)	102.68 ± 59.36	26 to 541
HDL Cholesterol (mg/dL)	51.78 ± 13.65	23 to 102
Total Cholesterol (mg/dL)	193.19 ± 40.25	51 to 330
LDL Cholesterol (mg/dL)	122.23 ± 34.41	32 to 262
TSH (mU/L)	1.97 ± 1.23	0.29 to 12.6
FT3 (pg/mL)	3.03 ± 0.41	2.01 to 4.6
FT4 (pg/mL)	10.41 ± 1.36	6.8 to 14.7
Vitamin D (ng/mL)	20.22 ± 7.98	4 to 50.4
Uric Acid (mg/dL)	4.62 ± 1.53	1.4 to 12.3
Creatinine (µmol/L)	65.64 ± 29.36	1 to 131
GOT** (U/L)	21.3 ± 8.87	5.2 to 76
GPT** (U/L)	32.87 ± 16.85	9 to 123
GGT** (U/L)	29.7 ± 28.24	5 to 290
Sideremia (mcg/dL)	80.15 ± 29.33	20 to 171
Sodium (mmol/L)	140.47 ± 2.17	135 to 146
Potassium (mmol/L)	4.27 ± 0.32	3.5 to 5.6
RBC** (10^12^/L)	4.93 ± 0.55	3.81 to 10.8
WBC** (10^3^/µL)	7.13 ± 1.74	3.26 to 12.7
Platelets (10^3^/µL)	262.94 ± 64.31	101 to 568

* Numeric variables are shown as mean ± standard deviation (SD) while categorical variables are shown as proportion (%). A reference range was included for all variables. ** BMI (Body Mass Index); WC (Waist circumference); SBP (Systolic Blood Pressure); DBP (Diastolic Blood Pressure); FBG (Fasting Blood Glucose); HOMA-IR (Homeostasis Model Assessment – Insulin Resistance); GOT (Glutamic Oxaloacetic Transaminase); GPT (Glutamic Pyruvic Transaminase); GGT (Gamma-Glutamyl Transpeptidase); RBC (Red Blood Cells); WBC (White Blood Cells).

**Table 2:** Spearman’s correlation test between both Vitamin D and Platelet number with all characteristics analyzed.

Variables	Vitamin D (*p*-Value)	Direction (Rho)	Platelet (*p*-Value)	Direction (Rho)
Age (years)	0.692	POSITIVE	0.032	NEGATIVE
BMI (kg/m^2^)	<0.01	NEGATIVE	0.087	POSITIVE
WC (cm)	0.001	NEGATIVE	0.338	POSITIVE
SBP (mm Hg)	0.280	NEGATIVE	0.411	NEGATIVE
DBP (mm Hg)	0.004	NEGATIVE	0.479	POSITIVE
FBG (mg/dL)	0.394	NEGATIVE	0.605	POSITIVE
Insulin (mg/dL)	0.006	NEGATIVE	0.001	POSITIVE
HOMA-IR	0.005	NEGATIVE	0.001	POSITIVE
Triglycerides (mg/dL)	0.163	NEGATIVE	0.747	POSITIVE
HDL Cholesterol (mg/dL)	0.720	POSITIVE	0.806	POSITIVE
Total Cholesterol (mg/dL)	0.613	NEGATIVE	0.253	POSITIVE
LDL Cholesterol (mg/dL)	0.613	NEGATIVE	0.074	POSITIVE
TSH (mU/L)	0.456	NEGATIVE	0.039	POSITIVE
FT3 (pg/mL)	0.396	NEGATIVE	0.442	POSITIVE
FT4 (pg/mL)	0.394	NEGATIVE	0.561	POSITIVE
Vitamin D (ng/mL)	NA	NA	0.045	NEGATIVE
Uric Acid (mg/dl)	0.218	NEGATIVE	0.031	NEGATIVE
Creatinine (µmol/L)	0.105	POSITIVE	0.176	NEGATIVE
GOT (U/L)	0.759	NEGATIVE	0.106	NEGATIVE
GPT (U/L)	0.346	NEGATIVE	0.635	NEGATIVE
GGT (U/L)	0.153	NEGATIVE	0.865	POSITIVE
Sideremia (mcg/dL)	0.704	NEGATIVE	0.084	NEGATIVE
Sodium (mmol/L)	0.600	POSITIVE	0.118	NEGATIVE
Potassium (mmol/L)	0.565	POSITIVE	0.322	POSITIVE
RBC (10^12^/L)	0.085	NEGATIVE	0.530	NEGATIVE
WBC (10^3^/µL)	0.077	NEGATIVE	<0.01	POSITIVE
Platelets (10^3^/µL)	0.045	NEGATIVE	NA	NA

**Table 3:** First linear regression model with platelet count as the independent variable.

	Coefficient	*p*-Value	Confidence Interval 95%
Vitamin D (ng/mL)	−0.78	0.07	−1.64 to 0.08
Age (years)	−0.21	0.42	−0.74 to 0.31
HOMA index	1.39	0.41	−1.96 to 4.76
TSH (mU/L)	4.2	0.13	−1.34 to 9.74
Uric acid (mg/dL)	−5.12	0.03	−9.87 to −0.37
WBC (10^3^/µL)	12.29	<0.01	8.26 to 16.31

**Table 4:** Second linear regression model with platelet count as the independent variable.

	Coefficients	*p*-Value	Confidence Interval 95%
Vitamin D (ng/mL)	−0.65	0.13	−1.51 to 0.2
Gender (male)	−31.95	<0.01	−49.77 to −14.13
BMI (kg/m^2^)	−0.42	0.52	−1.72 to 0.88
Age (years)	−0.1	0.68	−0.63 to 0.42
HOMA index	2.34	0.17	−1.08 to 5.78
TSH (mU/L)	3.26	0.24	−2.21 to 8.74
Uric acid (mg/dL)	−0.25	0.92	−5.65 to 5.14
WBC (10^3^/µL)	12.68	<0.01	8.67 to 16.69

## Abbreviations

BMI (Body Mass Index), HOMA-IR (Homeostasis Model Assessment of Insulin Resistance), 25(OH)D (25-hydroxyvitamin D), CVDs (Cardiovascular Diseases), BP (Blood Pressure), WC (Waist Circumference), DBP (Diastolic Blood Pressure)
